# Effects of national volume-based procurement policy on the usage and expenditure of platinum antineoplastic drugs in China: an interrupted time series study

**DOI:** 10.3389/fphar.2025.1593099

**Published:** 2025-08-25

**Authors:** Xihui Yu, Hailing Lin, Bin Li, Hongbo Fu, Yaofeng Zhang

**Affiliations:** Department of Pharmacy, The Second Affiliated Hospital of Shantou University Medical College, Shantou, Guangdong, China

**Keywords:** national volume-based drug procurement, platinum-based chemotherapy drugs, interrupted time series, gastrointestinal tumors, oxaliplatin

## Abstract

**Objective:**

Digestive tract tumors are the common tumors in China. Platinum-based chemotherapy drugs, especially oxaliplatin, play an important role in the treatment of gastrointestinal tumors. Starting from October 2021, the fifth national centralized volume-based procurement (NCVBP) was implemented in China to reduce the price of oxaliplatin. However, the effect of the policy on platinum antineoplastic drugs is uncertain. This study aims to explore the impact of NCVBP policy on the usage and expenditure of platinum antineoplastic drugs in China.

**Methods:**

Oxaliplatin and alternative drugs were used as objects of study to explore the impact of NCVBP policy from the first quarter of 2017 to the second quarter of 2024, while October 2021 was the implementation time point of the policy. Quarterly data was collected from the Chemical Drug Terminal of China’s Public Medical Institutions database in 1,805 sample public hospitals and more than 3,000 urban public hospitals in China. Descriptive analysis was conducted using purchase volume, expenditure and daily cost as variables. Interrupted time-series (ITS) analysis was applied to further analyze the effect of NCVBP policy on the medicines under study.

**Results:**

The average volume of bid-winning drug of oxaliplatin increased by 623.66% after the implementation of the policy, and its expenditure increased by 7.92%. The decline in price had promoted the increase in consumption, and the policy had realized the exchange of price for quantity. After the implementation of NCVBP, a significant increase was associated with the volume of bid-winning drugs (P < 0.001) and the trend change was statistically significant (P < 0.001), with an upward trend. The volume of non-winning drugs and the branded drug decreased immediately after policy intervention (P < 0.001), but there was no obvious downward trend change following. ITS analysis indicated that there were no significant differences in level and trend changes for expenditure of in alternative drugs.

**Conclusion:**

The policy had an influence on promotion of generic substitution and the decrease in expenditures for platinum antineoplastic drugs while ensuring the usage of drugs. The third-generation platinum antineoplastic medications introduced into the fifth NCVBP policy had no major influence on the use of first- and second-generation platinum antineoplastic treatments. Although the general situation for alternative pharmaceuticals was largely steady, with no expected increase in costs and expenditures, monitoring of alternative drugs was required.

## 1 Introduction

Cancer is a serious societal, public health, and economic challenge in the twenty-first century, accounting for roughly one in six deaths (16.8%) and one in four deaths (22.8%) from noncommunicable diseases globally (Bray et al., 2024). According to the current statistics provided by the Journal of the National Cancer Center, digestive tract cancers are the prevalent tumors in China, among which the incidence of gastric cancer, liver cancer, and colorectal cancer are in the top five (Han et al., 2024). In 2022, there were 517,100 new cases and 240,000 deaths of colorectal cancer in China. There were 367,700 new cases and 316,500 deaths of liver cancer. There were 358,700 new cases and 260,400 deaths of the gastrointestinal tract cancer. Gastrointestinal cancers such as intestinal and liver cancer are increasingly common and impose a heavy financial burden on most patients. Consequently, society is now deeply concerned with finding ways to ensure that no one has to abandon treatment simply because they cannot afford it. At presently, chemotherapy is one of the key ways for the treatment of malignant tumors, and platinum medicines have substantial application value in the treatment of malignant tumors. As the fundamental chemotherapeutic medication of digestive tumor treatments, it is extensively utilized in bowel cancer and liver cancer. Clinicians widely use platinum-based medicines in anti-tumor therapy. The drugs bind directly to tumor-cell DNA chains and disrupt their replication (Yasui et al., 2014; Nechay et al., 2023). It may be claimed that the usage of this medicine is closely connected to the treatment of digestive system malignancies. Clinically, drug-herb interactions can also be considered to reduce the side effects of platinum-based drugs and enhance their efficacy (Zhang et al., 2015; Li et al., 2018). Prodrug-based approaches are also gaining traction as an effective means to lessen the toxic side effects of chemotherapy drugs (Feng et al., 2024). There are five kinds of platinum medications routinely used in clinical practice, including the first generation of cisplatin, the second generation of carboplatin, nedaplatin and the third generation of oxaliplatin and loplatin. The nephrotoxicity, gastrointestinal response and neurotoxicity of cisplatin, the first-generation platinum medication, were severe (Shahid et al., 2018). After further improvement, the nephrotoxicity and gastrointestinal reaction of carboplatin and nedaplatin were greatly decreased, and hematological toxicity was the predominant adverse reaction of the second-generation platinum medication (Zheng et al., 2024). Nevertheless, platinum drug resistance is an important problem in platinum chemotherapy. Cisplatin and carboplatin eventually create drug resistance throughout therapy. Therefore, the third generation of platinum therapeutic medication oxaliplatin was created. The mechanism of action of oxaliplatin is comparable to cisplatin, without causing cross-resistance with cisplatin or carboplatin (Zhang et al., 2022). The major bad impact of oxaliplatin is neurotoxicity, although it should be highlighted that the predominant adverse effect of loplatin is still hematological toxicity (Shahlaei et al., 2024). Oxaliplatin has been found to be at least as efficacious as cisplatin for gastric cancer, with less toxicity and a better tolerability profile (Wang et al., 2019; Zhang et al., 2019).

China has been aggressively regulating the fast-expanding medical expenses and relieving the financial difficulties of medical treatment for patients. Centralized procurement is a group medication purchase approach overseen by the government, a consortium of medical institutions, or a third party. Its purpose is to manage medication procurement and sales, lower the societal burden of medical expenditures, and optimize industry competitive patterns. In November 2018, the General Office of the State Council issued the “4 + 7”national centralized volume-based procurement (NCVBP) policy, which is based on centralized purchasing employing the “volume for price” method. The core of this program is the trade-off between increased purchase quantities and cheaper pricing. First, the public medical institution sends an agreed-upon procurement list of pharmaceuticals and volumes to the National Health Security Office, based on 60%–70% of the previous year’s drug consumption estimate. Second, the National Health and Family Planning Commission holds bidding and pricing discussions in which enterprises that pass the drug consistency test can participate. Finally, for each type of medicine, whichever company with the lowest price and the agreed-upon volume wins the bid (Qiu et al., 2025). The NCVBP is now in its 10th round, having expanded from the original “4 + 7”pilot cities to the entire country. China inaugurated the fifth NCVBP nationwide in October 2021. The price of oxaliplatin, which is included in the fifth NCVBP, has dropped drastically by 82.4%.

As an active study topic, the NCVBP policy’s impact on the use and spending of platinum-based anticancer agents remains unknown. Because of a lack of qualitative and quantitative evidence for the aforementioned consequences, we performed an exploratory study to assess the impact of the NCVBP policy on the use and cost of platinum antineoplastic drugs. This is a study to assess the effects of the NCVBP implementation on platinum-based medications, and the results of the empirical analysis will assist the government optimize the policy, therefore improving its efficacy.

## 2 Materials and methods

### 2.1 Data source and collection

The data in this paper come from the database of “Chemical Drug Terminal of China’s Public Medical Institutions” (https://www.menet.com.cn/menetDatabase/databaseHigh.html), which covers 1,805 sample public hospitals and more than 3,000 urban public hospitals across the country. We extracted the information of selected products and alternative drugs of platinum anti-tumor drugs from the first quarter of 2017 to the second quarter of 2024 (the four-quarters of each year are referred to as Q1, Q2, Q3 and Q4 in turn). The main contents of the data include drug name, company name, specifications, quarterly consumption and consumption amount. Since these data are based on the statistics of drug sales of hospitals for each quarter rather than the drug usage data for each individual patients, there is no data on the number of patients. It is certain that the sample hospitals collected each quarter remain unchanged. Thus, it controls any potential bias in the medication usage caused by any mergers, closures, or additions of hospitals.

**FIGURE 1:**
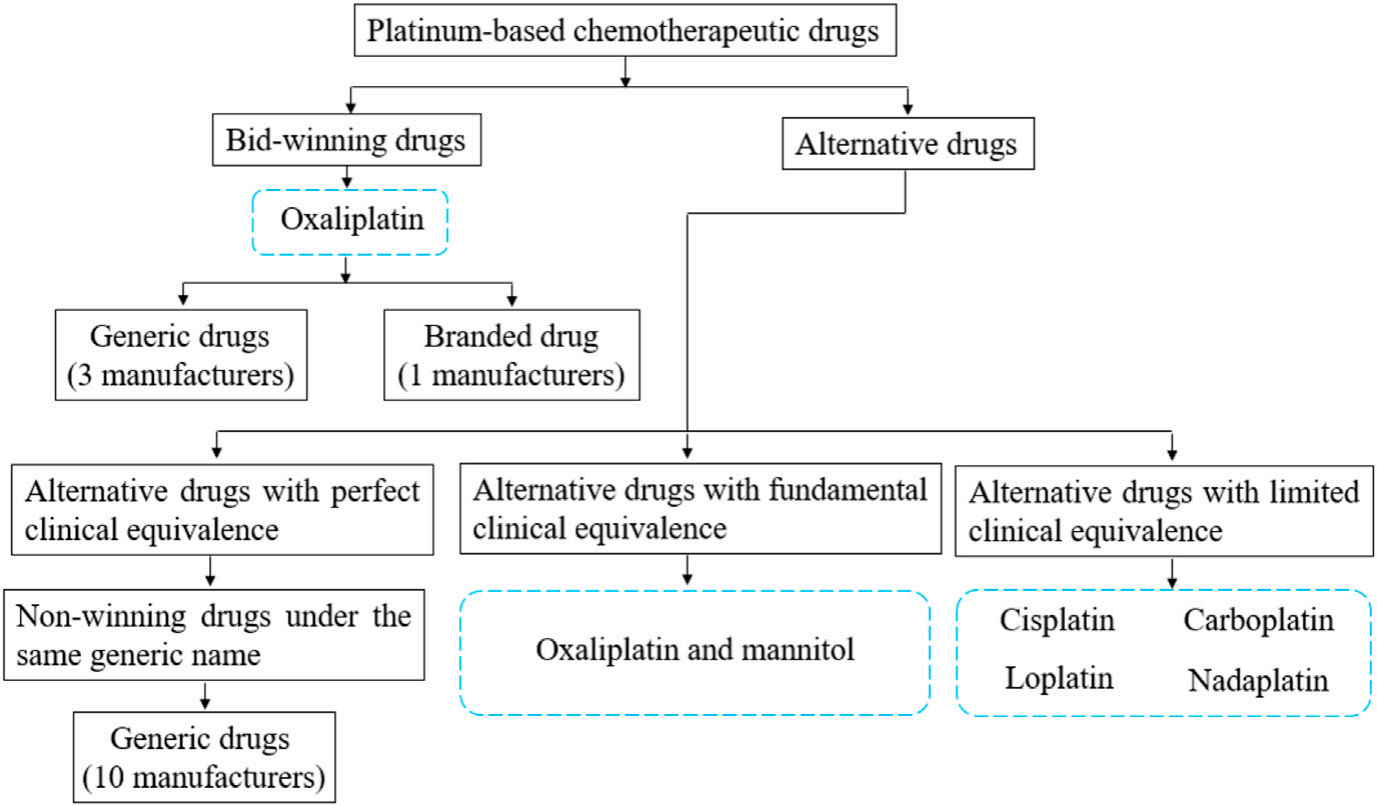
The classification of surveyed drugs and the relationship between bid-winning drugs and alternative drugs.

Alternative drugs are regarded as therapeutic equivalents with different active pharmaceutical ingredients but the same administration route. The National Healthcare Security Administration (NHSA) recently released the reference monitoring range of alternative drugs of NCVBP, which divided alternative drug varieties into three categories (Figure 1): alternative drug products with perfect clinical equivalence, alternative drug products with fundamental clinical equivalence, and alternative drug products with limited clinical equivalence (Wang et al., 2023a).

### 2.2 Data processing

We used the specified daily dosage (DDD) as the unit of measurement to standardize drug consumption and guarantee similar usage of various medications. The recommended adult dosage found in the manufacturer’s instructions was used to calculate the DDD value of certain drugs that were unable to be coded in WHO’s system. Defined Daily Doses (DDDs), which were computed by dividing the volume of sales data by the DDD, were used to measure volume. In order to enhance the reference value of this research in other countries, we convent Chinese Yuan (CNY) to United States dollar (USD) to report the expenditure statistics by the Campbell-Cochrane converter (https://eppi.ioe.ac.uk/costconversion/). Defined Daily Drug Cost (DDDc), which was calculated as the Expenditures/DDDs, was used to evaluate the daily cost.

### 2.3 Statistical analysis

By analyzing the utilization of policy-related drugs, the sustainability of the policy can be evaluated. To analyze trends in the volume, expenditures, and price of policy-related drugs, we performed a single-group Interrupted Time Series (ITS) from 2017 Q1 to 2024 Q2. In this study, we determined 2021 Q4 as the implementation time point of the NCVBP of platinum-based chemotherapeutic drugs (the fifth NCVBP). To estimate changes in the levels and trends of each outcome variable after NCVBP, the ITS model formula can be expressed as follows:

$$Y_{t}=\beta_{0}+\beta_{1}\;*\;{time}_{t}+\beta_{2}\;*\;level+\beta_{3}\;*\;trend+e_{t}$$ (1)

where Y_t_ refers to outcome variables (volume, expenditures, or DDDc) in month t; time_t_ is a continuous variable representing time trend; level represents a dummy variable for the policy intervention; the trend is an interaction term between time and level, and the e_t_ is an estimate of the random error term.

In this model, β_0_ estimates the baseline level at time = 0. β_1_, β_2_ and β_3_ represent the trend prior to intervention, the level change (immediate effect) that occurs immediately after the intervention, and the slope change (sustained effect) caused by the intervention, respectively. Estimates of the regression parameters and their variances from model (Equation 1) can be obtained from fitting a segmented linear regression model using ordinary least squares (OLS). β_0_ is the intercept of the linear regression line before the policy intervention. β_1_ is the slope of the linear regression line before the policy intervention. β_2_ is the change in the Y_t_-value at the policy intervention point between the linear regression lines before and after the policy intervention. β_3_ is the difference in slope between the linear regression lines before and after the policy intervention. A larger absolute value of β_2_ indicates that the instantaneous impact of the policy is more obvious. The greater the absolute value of β_3_, the more pronounced the policy’s impact on long-term trends.

We used the Augmented Dickey–Fuller (ADF) test to examine the stationarity of residuals of the model. Durbin-Watson (DW) test was used to check the serial autocorrelation. The DW-statistic can range between zero and four, with values close to two indicating no autocorrelation. As a form of generalized least squares (GLS), Prais-Winsten is used to control serial autocorrelation of the original model. If there is serial autocorrelation, Prais-Winsten estimation was used to correct the first-order serial correlation error. Sensitivity analysis was also assessed by robust regression (Huber regression and Tukey’s biweight), which are used to evaluate robustness of our results.

Although the recommendation by the Effective Practice and Organization of Care (EPOC) Cochrane Group suggests that ITS designs should have at least three time points per period for inclusion, methodological literature examined in a scoping review show that a minimum of eight time points per period is required to gain sufficient power in estimating the regression coefficients (Ewusie et al., 2020). In this study, 19 and 11 time points were used to ITS analysis before and after the policy intervention, respectively. This study meets the requirements for sample size.

## 3 Results

### 3.1 General characteristics

Following the adoption of the policy, the average volume of the bid-winning medicine grew by 623.66%, and its expenditure increased by 7.92%, as indicated in Table 1. Nonetheless, there was a clear 83.93% drop in the amount of non-winning product purchases, and the associated spending dropped by 87.92%. Generic medicine use and spending both climbed somewhat by 23.38% and 16.34%, respectively. The branded drug’s use and cost dropped by 25.97% and 38.47%, respectively. While the equivalent spending fell by 2.44%, the number of alternative drug purchases climbed by 28.94%.

**TABLE 1:** The average quarterly data of platinum anti-tumor drugs before and after the fifth NCVBP.

Outcome indicators	Categories	Before the 5th NCVBP	After the 5th NCVBP	Rate of increase (%)
Average quarterly DDDs (×10^4^)	Bid-winning drug	113.01	817.81	623.66
	Non-winning drugs	682.75	109.73	−83.93
	The branded drug	110.03	81.45	−25.97
	Generic drugs	685.73	846.08	23.38
	Alternative drugs	298.02	384.26	28.94
Average quarterly expenditure (×10^4^ USD)	Bid-winning drug	13179.49	14223.84	7.92
	Non-winning drugs	5774.76	697.5	−87.92
	The branded drug	13009.71	8005.32	−38.47
	Generic drugs	5944.53	6916.02	16.34
	Alternative drugs	3346.63	3265.07	−2.44
Average quarterly DDDc (USD)	Bid-winning drug	119.27	17.48	−85.34
	Non-winning drugs	8.71	6.41	−26.41
	The branded drug	120.1	98.2	−18.23
	Generic drugs	8.91	8.18	−8.19
	Alternative drugs	16.26	15.25	−6.21

### 3.2 ITS analysis of changes in volumes

**FIGURE 2:**
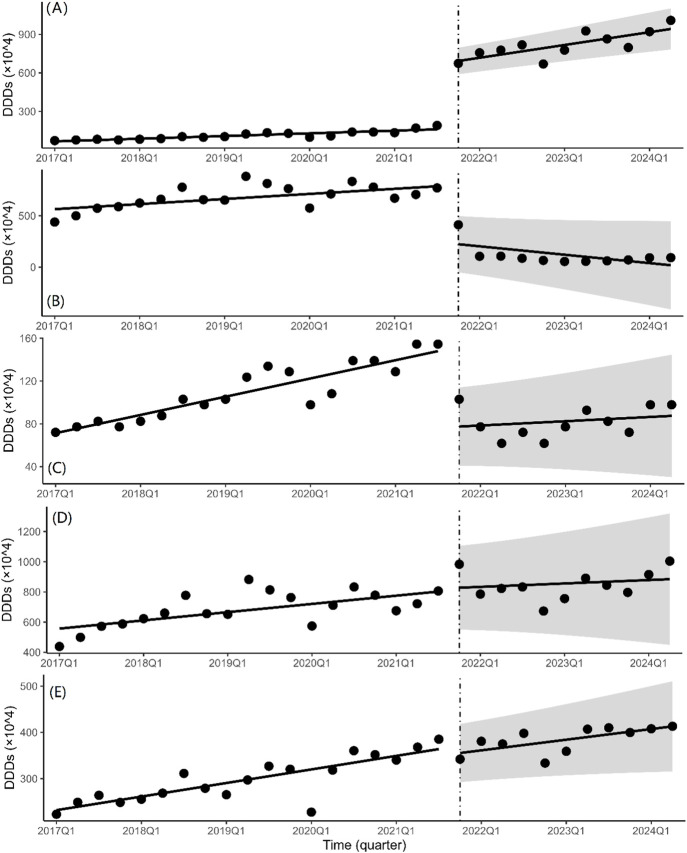
Quarterly trends in the volumes of platinum-based chemotherapeutic drugs. Interrupted time series analysis of volume changes of **(A)** bid-winning drugs, **(B)** non-winning drugs, **(C)** the branded drug, **(D)** generic drugs and **(E)** alternative drugs.

In the ITS analysis, DDDs was used as the indicator for the volume of drugs. The quarterly trends in the volume of drugs are displayed in Figure 2 and Table 2 presents the results of ITS analysis for procurement volume. The volume of bid-winning medications increased significantly (*P* < 0.001) following the introduction of NCVBP, and the trend change was statistically significant (*P* < 0.001) and showed an increasing trend. Following the policy action, the volume of branded and non-winning medications dropped quickly (*P* < 0.001), but there was no discernible downward trend shift. Every quarter, the instantaneous level of DDDs for generic medications rose by 19.73 ten thousand DDDs (*P* = 0.819). Every quarter, there was a 14.27 ten thousand DDDs drop in the immediate level of alternative medications (*P* = 0.485). There was no statistically significant difference in the downward trend change between generic and alternative medications.

**TABLE 2:** The results of interrupted time series analysis for DDDs (×10^4^), expenditure (×10^4^ USD), and DDDc (USD).

Categories	Products	Constant			Secular trend			Level change			Trend change			Durbin-watson value	Dickey–fuller statistics	R-squared
		β_0_ (95%CI)	Std. error	*P*	β_1_ (95%CI)	Std. error	*P*	β_2_ (95%CI)	Std. error	*P*	β_3_ (95%CI)	Std. error	*P*			
DDDs (×10^4^)	Bid-winning drugs	60.71 (19.12, 102.29)	20.23	<0.001	5.23 (1.58, 8.88)	1.77	0.007	508.01 (439.84, 576.19)	33.17	<0.001	19.72 (10.65, 28.79)	4.41	<0.001	1.979	−7.8221**	0.987
	Non-winning drugs	551.44 (437.42, 665.45)	55.47	<0.001	12.37 (2.48, 22.25)	4.81	0.016	−542.52 (−714.59, −370.46)	83.71	<0.001	−32.79 (−57.15, −8.43)	11.85	0.010	1.804	−4.0303*	0.867
	The branded drug	67.05 (51.90, 82.20)	7.37	<0.001	4.26 (2.94, 5.57)	0.64	<0.001	−71.49 (−94.64, −48.34)	11.26	<0.001	−3.26 (−6.50, −0.01)	1.58	0.049	1.803	−4.4855**	0.715
	Generic drugs	543.60 (428.08, 659.12)	56.2	<0.001	13.63 (3.61, 23.65)	4.87	0.010	19.73 (−155.35, 194.82)	85.18	0.819	−7.97 (−32.67, 16.74)	12.02	0.513	1.729	−4.4233**	0.353
	Alternative drugs	224.71 (199.45, 249.97)	12.29	<0.001	7.33 (5.11, 9.55)	1.08	<0.001	−14.27 (−55.71, 27.17)	20.16	0.485	−1.57 (−7.08, 3.94)	2.68	0.485	2.000	−5.8419**	0.834
Expenditure (×10^4^ USD)	Bid-winning drugs	9054.80 (7296.22, 10813.38)	855.54	<0.001	409.99 (257.08, 562.90)	74.39	<0.001	−3951.04 (−6662.47, −1239.61)	1319.09	0.006	−173.05 (−550.58, 204.47)	183.66	0.355	1.775	−4.8217**	0.476
	Non-winning drugs	6433.37 (5826.08, 7040.66)	295.44	<0.001	−67.41 (−120.41, −14.40)	25.79	0.015	−3743.46 (−4705.08, −2781.84)	467.82	<0.001	−47.12 (−178.32, 84.08)	63.83	0.467	1.843	−4.9111**	0.948
	The branded drug	9372.70 (7818.03, 10927.36)	756.33	<0.001	359.63 (224.12, 495.13)	65.92	<0.001	−8121.05 (−10558.32, −5683.79)	1185.71	<0.001	−357.40 (−692.45, −22.35)	163.00	0.038	1.842	−4.7943**	0.788
	Generic drugs	6064.91 (5164.48, 6965.34)	438.05	<0.001	−11.38 (−89.12, 66.36)	37.82	0.766	279.56 (−1041.34, 1600.46)	642.61	0.667	145.07 (−46.17, 336.31)	93.04	0.131	1.709	−4.1608**	0.329
	Alternative drugs	2999.39 (2727.36, 3271.43)	132.34	<0.001	34.79 (10.90, 58.68)	11.62	0.006	−350.74 (−801.43, 99.94)	219.26	0.122	−42.21 (−101.73, 17.31)	28.96	0.157	2.044	−6.6343**	0.333
DDDc (USD)	Bid-winning drugs	137.96 (129.31, 146.62)	4.21	<0.001	−1.99 (−2.71, −1.27)	0.35	<0.001	−74.85 (−84.02, −65.68)	4.46	<0.001	1.05 (−0.68, 2.78)	0.84	0.224	1.837	−2.5283	0.978
	Non-winning drugs	12.54 (10.61, 14.47)	0.94	<0.001	−0.33 (−0.47, −0.18)	0.07	<0.001	−0.49 (−1.67, 0.69)	0.57	0.398	0.34 (0.10, 0.67)	0.16	0.044	1.742	−2.2936	0.834
	The branded drug	135.22 (129.26, 141.18)	2.90	<0.001	−1.54 (−2.05, −1.03)	0.25	<0.001	0.36 (−7.58, 8.31)	3.86	0.926	0.32 (−0.93, 1.56)	0.6	0.606	1.929	−2.7441	0.927
	Generic drugs	12.91 (9.29, 16.53)	1.76	<0.001	−0.24 (−0.45, −0.04)	0.10	0.022	−1.61 (−2.70, −0.53)	0.53	0.005	0.37 (−0.03, 0.76)	0.19	0.066	1.290	−1.9117	0.692
	Alternative drugs	17.59 (16.76, 18.41)	0.40	<0.001	−0.13 (−0.19, −0.06)	0.03	<0.001	0.24 (−0.75, 1.22)	0.48	0.627	0.08 (−0.09, 0.24)	0.08	0.367	2.113	−2.2648	0.920

*P < 0.05; **P < 0.01.

### 3.3 ITS analysis of changes in expenditure

**FIGURE 3:**
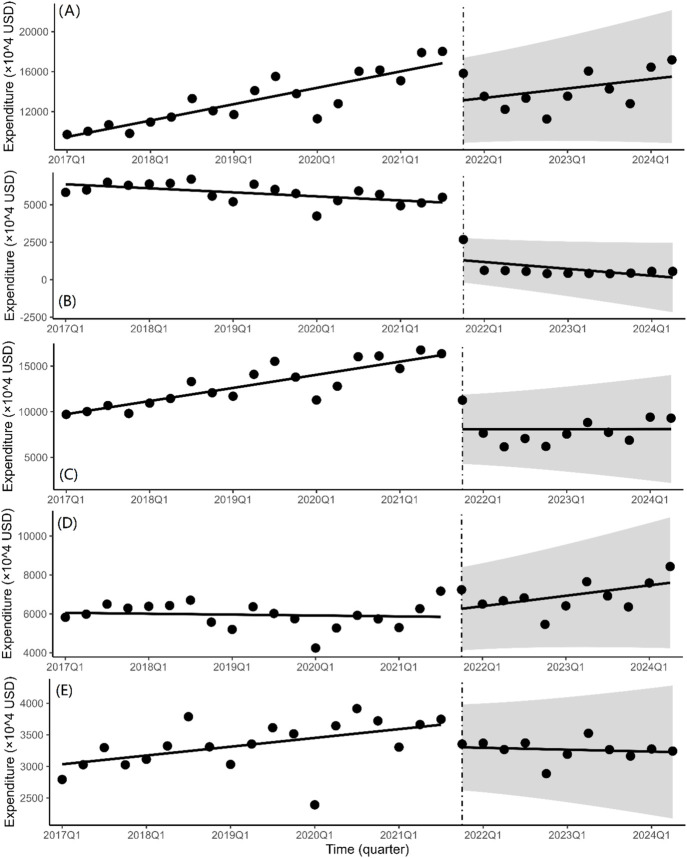
Quarterly trends in the expenditure of platinum-based chemotherapeutic drugs. Interrupted time series analysis of expenditure changes of **(A)** bid-winning drugs, **(B)** non-winning drugs, **(C)** the branded drug, **(D)** generic drugs and **(E)** alternative drugs.

The total quarterly expenditure of bid-winning medications (Figure 3A) was drastically decreased by 39.51 million USD on the instantaneous level shift following the fifth NCVBP policy (*P* = 0.006). There is a 1.73 million USD decline in the quarterly spending trend (*P* = 0.355). With a significant statistical difference (*P* < 0.001), the immediate level change in purchasing expenditure after the policy is 37.43 million USD for non-winning pharmaceuticals (Figure 3B) and 81.21 million USD for branded drugs (Figure 3C). We discovered that the instantaneous level shift caused a modest rise in the cost of purchasing generic medications (Figure 3D) (β_2_ = 2.80 × 10^6^, *P* = 0.667). The instantaneous level change for alternative medications did not vary statistically significantly (Figure 3E) (β_2_ = −3.51 × 10^6^, *P* = 0.122).

### 3.4 ITS analysis of changes in the daily cost

**FIGURE 4:**
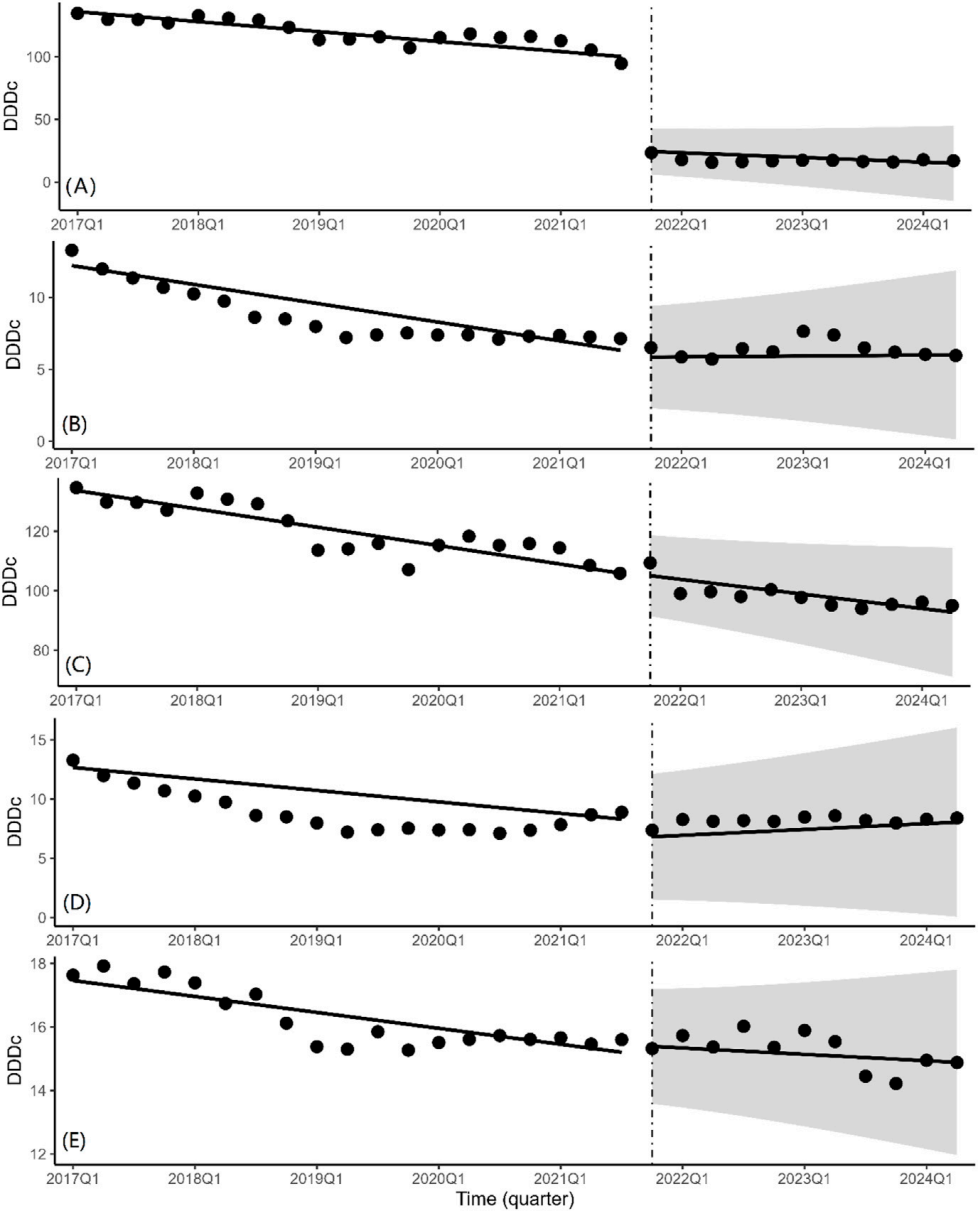
Quarterly trends in the daily cost of platinum-based chemotherapeutic drugs. Interrupted time series analysis of daily cost changes of **(A)** bid-winning drugs, **(B)** non-winning drugs, **(C)** the branded drug, **(D)** generic drugs and **(E)** alternative drugs.

According to the findings of the ITS analysis (Figure 4; Table 2), the DDDc of the bid-winning drug (β_1_ = −1.99, *P* < 0.001), non-winning drug (β_1_ = −0.33, *P* < 0.001), and the branded drug (β_1_ = −1.54, *P* < 0.001) clearly exhibited a substantial decreasing tendency prior to the introduction of NCVBP. The DDDc of bid-winning medications abruptly decreased when the policy was put into effect (β_2_ = −74.85, *P* < 0.001). Other items showed statistically significant differences and no significant modifications.

### 3.5 Sensitivity analysis

Huber regression and Tukey’s biweight methods typically for the linear regression model that are insensitive to outliers and possibly high leverage points. The results of ITS analysis for DDDs (×10^4^), expenditure (×10^4^ USD), and DDDc (USD) in Huber regression model and Tukey’s biweight regression model are shown in Supplementary Table S1 and Supplementary Table S2, respectively. We compared trend changes indicator (β_3_) in different models. The direction of β_3_ in most cases remains consistent across the three models. As shown in Table 3, the direction of β_3_ remained consistent in the three models and *P* value was less than 0.05 in the DDDs of bid-winning drugs and non-winning drugs. The fifth NCVBP policy has a significantly influence on the increase in the usage of bid-winning drugs and the decrease in the usage of non-winning drugs in oxaliplatin. The result is robust.

**TABLE 3:** Comparison trend changes indicator (β_3_) of different models through sensitivity analysis.

Categories	Products	Model	β_3_	Std. error	*P*
DDDs (×10^4^)	Bid-winning drugs	GLS	19.72	4.41	<0.001
		Huber’s Method	18.78	3.05	<0.001
		Tukey’s Biweight	21.4	2.83	<0.001
	Non-winning drugs	GLS	−32.79	11.85	0.010
		Huber’s Method	−18.58	6.83	0.011
		Tukey’s Biweight	−14.56	6.68	0.04
	The branded drug	GLS	−3.26	1.58	0.049
		Huber’s Method	−2.25	1.28	0.089
		Tukey’s Biweight	−1.31	1.22	0.295
	Generic drugs	GLS	−7.97	12.02	0.513
		Huber’s Method	−4.04	10.52	0.704
		Tukey’s Biweight	−2.89	10.80	0.791
	Alternative drugs	GLS	−1.57	2.68	0.485
		Huber’s Method	−2.15	2.11	0.316
		Tukey’s Biweight	−2.38	1.94	0.231
Expenditure (×10^4^ USD)	Bid-winning drugs	GLS	−173.05	183.66	0.355
		Huber’s Method	−112.87	156.26	0.477
		Tukey’s Biweight	−123.31	161.33	0.452
	Non-winning drugs	GLS	−47.12	63.83	0.467
		Huber’s Method	19.38	43.92	0.663
		Tukey’s Biweight	48.97	39.56	0.227
	The branded drug	GLS	−357.40	163.00	0.038
		Huber’s Method	−233.08	127.65	0.079
		Tukey’s Biweight	−163.96	125.67	0.203
	Generic drugs	GLS	145.07	93.04	0.131
		Huber’s Method	117.91	67.95	0.095
		Tukey’s Biweight	123.23	74.15	0.109
	Alternative drugs	GLS	1.05	0.84	0.224
		Huber’s Method	−53.66	18.37	0.007
		Tukey’s Biweight	−58.09	19.03	0.005
DDDc (USD)	Bid-winning drugs	GLS	1.05	0.84	0.224
		Huber’s Method	1.28	0.45	0.009
		Tukey’s Biweight	1.26	0.46	0.010
	Non-winning drugs	GLS	0.34	0.16	0.044
		Huber’s Method	0.29	0.08	0.002
		Tukey’s Biweight	0.30	0.09	0.003
	The branded drug	GLS	0.32	0.6	0.606
		Huber’s Method	0.54	0.39	0.181
		Tukey’s Biweight	0.53	0.42	0.219
	Generic drugs	GLS	0.37	0.19	0.066
		Huber’s Method	0.33	0.09	<0.001
		Tukey’s Biweight	0.40	0.08	<0.001
	Alternative drugs	GLS	0.08	0.08	0.367
		Huber’s Method	0.04	0.05	0.507
		Tukey’s Biweight	0.04	0.06	0.478

## 4 Discussion

The collection policy has a significant impact on the usage of platinum-based antitumor medicines. This program can successfully limit medication costs while simultaneously encouraging domestic substitution. These developments are helpful in providing patients with better and more cost-effective treatment alternatives. Oxaliplatin is a third-generation platinum-based chemotherapeutic medication with low toxicity and high tolerance (Capdevila et al., 2008). It is widely utilized in the treatment of gastrointestinal malignancies such as gastric cancer, colorectal cancer, and liver cancer, making it one of the most often used anticancer medications in clinical practice. The majority of patients, particularly those with colorectal cancer, select oxaliplatin as part of their treatment. The combination of oxaliplatin with 5-fluorouracil (5-FU)/leucovorin (LV) has been shown to considerably improve antitumor activity, increasing median progression-free survival from 6.1 to 8.7 months in 5-FU/LV treated and oxaliplatin/5-FU/LV treated patients, respectively (McQuade et al., 2017). The medicine is suitable for a wide spectrum of patients, including neoadjuvant therapy for preoperative patients, adjuvant therapy for postoperative patients, and even tumor-reducing therapy for advanced-stage patients. For patients with early-stage colorectal cancer, using oxaliplatin-containing combination regimens in adjuvant chemotherapy can increase 5-year survival rates by 15%–20%. For advanced-stage patients, oxaliplatin-based combination chemotherapy can increase survival by 6–12 months. As a result, the American Society of Clinical Oncology (ASCO) Guidelines, the European Society for Medical Oncology (ESMO) Guidelines, and the Chinese Society of Clinical Oncology (CSCO) Guidelines all support this regimen as a routine treatment choice (Lieu et al., 2019; Argilés et al., 2020; Wang et al., 2024). As one of bid-winning drugs, the price of branded oxaliplatin has reduced from almost 2,000 CNY to roughly 300 CNY. The decrease was greater than 80%. This indicates that patients can save up to 60,000 CNY during a 6-month course of therapy using a biweekly treatment plan. The NCVBP strategy has demonstrated the ability to reduce medicine prices, which may benefit from the increased purchasing power generated by Chinese massive market size.

The average quarterly purchase volume of bid-winning drugs, oxaliplatin, increased by 623.66% after the NCVBP policy was implemented, while the daily cost decreased by 85.34%. Overall, the policy’s short-term effects were evident. The fifth NCVBP policy has a significantly influence on the increased volume and the decreased defined daily drug cost of bid-winning drugs. The price reduction of the bid-winning drug, oxaliplatin, had be conducive to reduce the burden and improved accessibility for patients with digestive tract tumors. The above results fully reflected the NCVBP policy’s impact on lowering the public burden of drugs and promoting the treatment of digestive tract tumors in China.

The ITS regression analysis results revealed a significant increase in the quarterly purchase volume and corresponding quarterly trend change of bid-winning drugs before and after the NCVBP policy, while the opposite was observed for non-winning and branded drugs, and the procurement volume of generic and alternative drugs remained stable. In the fifth NCVBP, four manufacturers qualified for the selection of bid-winning oxaliplatin pharmaceuticals. One of the bid-winning drugs was for a branded medicine. According to Figures 2A,C, the NCVBP policy had a greater impact on the procurement volume of generic bid-winning drugs than on branded bid-winning drugs. It demonstrates that the NCVBP strategy was beneficial for domestic substitution, which was identified as a way to improve medicine cost and accessibility. The NCVBP strategy has changed the drug market, with drug consumption shifting toward bid-winning pharmaceuticals and domestic replacement. A discrete choice experiment in China found that generic consistency evaluation (GCE) strongly affected patients’ approval of generic marketing strategies, with patients preferring greater reimbursement rates for generic medications (Zhang et al., 2024). In the NCVBP policy, generic drugs that have passed the GCE or the branded drug are eligible to be a candidate for bid-winning drugs, which means that the quality and efficacy of generic bid-winning drugs are consistent with the branded drug. In addition, the price advantage and high reimbursement rates of the bid-winning drugs have greatly promoted its procurement volume. We observed significant DDDc reductions of the bid-winning drugs in Figure 4A. With the continuous improvement of the quality of generic drugs through GCE in China, there was a downward trend in daily cost of platinum antineoplastic drugs prior to NCVBP, and the implementation of the policy resulted in further price reductions. On the whole, there was no significant change in the trend of the use of generic drugs in Figure 2D. This may be due to the small market share of the branded drug before the implementation of the policy, which made the overall volume of generic drugs increase not obvious after part of the volume was transferred from the branded drug. From Figures 2A,B,D, we found that the total volume of generic drugs kept a balance, and the increment of the bid-winning generic drugs was basically provided by the non-winning generic drugs. In the analysis of alternative drugs, there was not significant statistical difference in the trend of quarterly purchases. This result indicated that the NCVBP policy did not influence the prescription behavior of doctors on decreasing the usage of alternative drugs.

The ITS regression analysis findings of alternative medications revealed no significant differences. From Figure 3E, the ‘bypass effect’ was not seen in the study. The ‘bypass effect’ is a phenomenon in which the expenditure on reduced-price pharmaceuticals stabilizes or declines while the usage of non-price-reduced drugs increases dramatically (Wang et al., 2023b). In general principles for gastrointestinal cancer, platinum agents–oxaliplatin, carboplatin, or cisplatin–may reasonably be substituted for one another in some settings (Santos et al., 2024). The absence of a bypass effect with oxaliplatin might be attributed to its particular benefit. Oxaliplatin is a third-generation platinum compound having a broad antitumor impact both *in vitro* and *in vivo*, a higher safety profile than cisplatin, and no cross-resistance with cisplatin or carboplatin. Oxaliplatin is an appealing alternative to cisplatin due to its much-reduced neurotoxicity profile (Kolomeyevskaya et al., 2015). Although the general situation for alternative pharmaceuticals was largely steady, with no expected increase in costs and expenditures, monitoring of alternative drugs was required.

Tradeoffs of price-for-quantity are the characteristics and advantages of NCVBP. The relationship between price and quantity is one of the most important concepts in economics. When the price of a product decreases, the quantity of the product that is purchased will generally increase. National Healthcare Security Administration accelerates the reduction of drug prices through NCVBP and achieves macro-control. The combination of quantity guaranteed by the government department and the price concessions made by the pharmaceutical manufacturers has achieved a win-win situation. Furthermore, NCVBP has also promoted the generic substitution. Generic drugs provide cost-effective alternatives to brand-name medications, improving access to vital therapies, lowering healthcare costs, and increasing medication adherence for continuous treatment. The broad availability and utilization of generic substitution results in considerable cost reductions for patients, payers, and healthcare systems, allowing for more sustainable healthcare funding and resource allocation. Generic pharmaceuticals provide patients with safe and effective treatment alternatives by assuring therapeutic equivalence and regulatory compliance, instilling trust in the effectiveness and quality of medications. Physicians benefit from the wide variety of generic pharmaceuticals accessible, allowing them to make educated prescription decisions that emphasize patient wellbeing while controlling healthcare costs. Overall, the impact of NCVBP goes beyond economic benefits and includes enhanced healthcare affordability, accessibility, eventually increasing public healthcare sustainability.

Building effective medicine pricing policies is a challenging task in all high-, middle- and low-income countries. Different countries adopt different drug pricing policies to address the issue of high pharmaceutical prices. An ITS analysis showed that external price referencing (EPR) policy significantly reduced household pharmaceutical spending in Georgia (Gorgodze et al., 2025). The government set medicine prices using EPR in their country based on the prices of the same medicines in other countries (Babar, 2024). In South Korea, actual transaction price (ATP) was the most frequently occurring mechanism of price reduction in anticancer drugs (Kwon et al., 2021). This means that the maximum allowable price should be adjusted downward when displaying the gap between the list price and the ATP. In sub-Saharan Africa, the use of generic medicines is a strategy to reduce prices and ensure improved access (Koduah et al., 2022). The majority of European countries use a price-regulated system. In United Kingdom, the National Health Service (NHS) purchases most hospital medicines through competitive bidding (Rodwin, 2021). The NHS England Commercial Medicines Unit issues requests for bids and it usually seeks two or three suppliers nationally. However, it had less impact than expected. Once manufacturers understood that the department of health would routinely cut prices, they set high launch prices to deal with future price cuts. In addition, price cuts did not discourage physicians from substituting low-cost medicines with newer more expensive products. Mechanisms such as price cap regulation and reference pricing have generated price reductions of originator and generic medicines, but pharmaceutical manufacturers have no incentive to lower their prices without competition (Dylst and Simoens, 2010). In China, reimbursement-linked price negotiations have focused on anticancer drugs that offer greater clinical value (Zhou et al., 2024). In the fifth NCVBP, the normalized unit price (NUP), a tool for setting a limit price, is calculated by converting each declared price in the smallest specification and basic dosage form of the same product of medicine. The tender price submitted by pharmaceutical manufacturers must satisfy any one of the following three criteria to be eligible for selection. (1) The NUP is no more than 1.8 times the lowest NUP of the same type. (2) The NUP has a reduction of more than or equal to 50.00% compared to the highest valid declared price (HVDP). The HVDP is determined by referring to the listed price in each province over a certain period. The lowest price, the average price or the most frequently occurring price is the reference standard. (3) The NUP is less than or equal to 0.1000 CNY (0.028 USD). The government adopts a last-place elimination mechanism to confirm more than one winning bidder. The winning enterprises will successively select the regions they want to supply to until all the regions have been selected. This prevents an excessive concentration of drug supply in a few manufacturers, thereby averting potential shortages. Compared with other countries, the fifth NCVBP also adopted the method of replacing branded drugs with generic drugs to achieve price reduction. The key differences of this policy lie in its unique pricing method, the guaranteed quantity, and the nationwide supply model involving multiple enterprises.

Compared with the bidding method used in other countries, the inclusion criteria of NCVBP for suppliers demonstrate a unique advantage. First, the NCVBP policy groups all products that share the same generic name into a single bidding pool. Any manufacturer that opts not to compete is automatically eliminated, guaranteeing that the bidding pool spans the entire market. This ensures that the offers are realistic and greatly reduces the risk of price manipulation by a handful of suppliers. Moreover, there are more than 50% volume in the same single bidding pool awarded to the bid-winning drugs. It means that manufacturers that either refuse to participate in the bidding process or fail to win the bid are basically shut out of the market during the policy cycle. This restricts non-winning manufacturers from continuing to be used by physicians at high prices. Although there is a risk of shortage for the bid-winning drug due to the sudden increase in usage, there is a backup selection mechanism for addressing the shortage risk. Although the fifth NCVBP has a beneficial influence on cost containment of patients, it is also necessary to acknowledge some possible unintended consequences, such as drug quality variability, therapeutic substitution, supply chain constraints. The prices of medicines cannot be reduced indefinitely and need to be kept at a reasonable level. At the same time, doctors need to make reasonable choices regarding the original and generic drugs based on the severity of the patient’s condition.

There were numerous possible limitations to this study. To begin, this study only looks at 1,805 sample public hospitals and more than 3,000 urban public hospitals across the country. Given the disparities in income levels and medication habits among areas, caution should be given when extrapolating the results. Second, other confounding variables, such as other reform programs, may impact drug use. However, we did not establish a control group to rule out other factors that may have influenced the outcomes of this study. Third, the findings of this study were based on drug spending data rather than prescription data. Although there is great agreement between spending data and usage data under NCVBP regulation, the two data sources may not perfectly match due to a stockpile of unutilized pharmaceuticals. Despite these limitations, this study conducted a thorough evaluation of the effect of NCVBP on platinum antineoplastic medications and revealed the influence of the NVBP policy on conventional cancer treatments. This study can help each person better understand and participate in the policy. At the same time, it will assist in the development, optimization, and implementation of future policies.

## 5 Conclusion

The findings presented in this study demonstrated that the introduction of the fifth NCVBP policy had an influence on reducing prices and boosting the usage of bid-winning oxaliplatin medications. The third-generation platinum antineoplastic medications introduced into the fifth NCVBP policy had no major influence on the use of first- and second-generation platinum antineoplastic treatments. Because digestive tract malignancies remain a major medical burden in China, this policy has improved the accessibility and affordability of oxaliplatin.

The study is the first to analysis the impact of national centralized volume-based procurement policy on platinum antineoplastic drugs under national drug sales records at 1,805 sample public hospitals and more than 3,000 urban public hospitals. At present, there is relatively little in-depth analysis of the impact of national drug policies on platinum-based anti-tumor drugs. This study has demonstrated the unique quantity-price trade-off feature of NCVBP and its effective reduction of medical costs. Furthermore, a thorough analysis was conducted to compare the fifth NCVBP policy with other policies. It can provide certain reference value for drug policy design of other countries.

## Data Availability

The original contributions presented in the study are included in the article/Supplementary Material, further inquiries can be directed to the corresponding author.

## Abbreviations

NCVBP, National Centralized Volume-based Procurement; NHSA, National Healthcare Security Administration; DDD, Defined Daily Dose; DDDs, Defined Daily Doses; CNY, Chinese Yuan; USD, United States dollar; OLS, ordinary least squares; GLS, generalized least squares; ADF, Augmented Dickey–Fuller; DW, Durbin-Watson; EPOC, Effective Practice and Organization of Care; EPR, external price referencing; ATP, actual transaction price; NUP, normalized unit price; HVDP, highest valid declared price; NHS, National Health Service; ITS, Interrupted Time Series; 5-FU, 5-fluorouracil; LV, Leucovorin; ASCO, American Society of Clinical Oncology; ESMO, European Society for Medical Oncology; CSCO, Chinese Society of Clinical Oncology; GCE, Generic Consistency Evaluation.
