# Supplementary material for: Effects of national volume-based procurement policy on the usage and expenditure of platinum antineoplastic drugs in China: an interrupted time series study
Source: Front Pharmacol. 2025 Aug 25;16:1593099. doi: 10.3389/fphar.2025.1593099 (PMC12414929; doi:10.3389/fphar.2025.1593099)
Supplement: Supplementary file 1 [file Table1.docx]

**Supplement Table 1.** The results of interrupted time series analysis for DDDs (×10^4^), expenditure (×10^4^ USD), and DDDc (USD) in Huber regression model

| **Categories** | **Products** | **Constant** | | | **Secular trend** | | | **Level change** | | | **Trend change** | | |
| --- | --- | --- | --- | --- | --- | --- | --- | --- | --- | --- | --- | --- | --- |
|  |  | **β_0_** | **Std. Error** | ***P*** | **β_1_** | **Std. Error** | ***P*** | **β_2_** | **Std. Error** | ***P*** | **β_3_** | **Std. Error** | ***P*** |
| **DDDs (×10^4^)** | Bid-winning drugs | 61.04 | 13.98 | <0.001 | 5.20 | 1.23 | <0.001 | 525.80 | 22.92 | <0.001 | 18.78 | 3.05 | <0.001 |
|  | Non-winning drugs | 545.47 | 31.32 | <0.001 | 13.13 | 2.74 | <0.001 | -674.82 | 51.35 | <0.001 | -18.58 | 6.83 | 0.011 |
|  | The branded drug | 65.83 | 5.85 | <0.001 | 4.51 | 0.51 | <0.001 | -85.12 | 9.60 | <0.001 | -2.25 | 1.28 | 0.089 |
|  | Generic drugs | 537.64 | 48.23 | <0.001 | 14.33 | 4.23 | 0.002 | -30.54 | 79.06 | 0.702 | -4.04 | 10.52 | 0.704 |
|  | Alternative drugs | 224.11 | 9.66 | <0.001 | 7.74 | 0.85 | <0.001 | -18.44 | 15.83 | 0.255 | -2.15 | 2.11 | 0.316 |
| **Expenditure (×10^4^ USD)** | Bid-winning drugs | 8979.99 | 716.55 | <0.001 | 430.13 | 62.85 | <0.001 | -4821.90 | 1174.67 | <0.001 | -112.87 | 156.26 | 0.477 |
|  | Non-winning drugs | 6436.57 | 201.40 | <0.001 | -61.45 | 17.66 | <0.001 | -4448.30 | 330.16 | <0.001 | 19.38 | 43.92 | 0.663 |
|  | The branded drug | 9278.63 | 585.39 | <0.001 | 377.86 | 51.34 | <0.001 | -9550.21 | 959.65 | <0.001 | -233.08 | 127.65 | 0.079 |
|  | Generic drugs | 6198.45 | 311.60 | <0.001 | -24.33 | 27.33 | 0.382 | 663.94 | 510.81 | 0.205 | 117.91 | 67.95 | 0.095 |
|  | Alternative drugs | 2956.92 | 84.23 | <0.001 | 43.18 | 7.39 | <0.001 | -436.14 | 138.09 | 0.004 | -53.66 | 18.37 | 0.007 |
| **DDDc (USD)** | Bid-winning drugs | 135.61 | 2.06 | <0.001 | -1.56 | 0.18 | <0.001 | -86.91 | 3.38 | <0.001 | 1.28 | 0.45 | 0.009 |
|  | Non-winning drugs | 11.57 | 0.39 | <0.001 | -0.29 | 0.03 | <0.001 | 0.26 | 0.64 | 0.683 | 0.29 | 0.08 | 0.002 |
|  | The branded drug | 134.76 | 1.80 | <0.001 | -1.4 | 0.16 | <0.001 | -5.00 | 2.95 | 0.102 | 0.54 | 0.39 | 0.181 |
|  | Generic drugs | 11.53 | 0.41 | <0.001 | -0.28 | 0.04 | <0.001 | 1.74 | 0.67 | 0.015 | 0.33 | 0.09 | <0.001 |
|  | Alternative drugs | 17.72 | 0.25 | <0.001 | -0.14 | 0.02 | <0.001 | 0.88 | 0.41 | 0.039 | 0.04 | 0.05 | 0.507 |

**Supplement Table 2.** The results of interrupted time series analysis for DDDs (×10^4^), expenditure (×10^4^ USD), and DDDc (USD) in Tukey's biweight regression model

| **Categories** | **Products** | **Constant** | | | **Secular trend** | | | **Level change** | | | **Trend change** | | |
| --- | --- | --- | --- | --- | --- | --- | --- | --- | --- | --- | --- | --- | --- |
|  |  | **β_0_** | **Std. Error** | ***P*** | **β_1_** | **Std. Error** | ***P*** | **β_2_** | **Std. Error** | ***P*** | **β_3_** | **Std. Error** | ***P*** |
| **DDDs (×10^4^)** | Bid-winning drugs | 61.12 | 12.99 | <0.001 | 5.20 | 1.14 | <0.001 | 526.4 | 21.30 | <0.001 | 21.40 | 2.83 | <0.001 |
|  | Non-winning drugs | 540.16 | 30.64 | <0.001 | 12.62 | 2.69 | <0.001 | -687.76 | 50.23 | <0.001 | -14.56 | 6.68 | 0.040 |
|  | The branded drug | 65.66 | 5.61 | <0.001 | 4.54 | 0.49 | <0.001 | -93.42 | 9.19 | <0.001 | -1.31 | 1.22 | 0.295 |
|  | Generic drugs | 537.76 | 49.51 | <0.001 | 14.26 | 4.34 | <0.001 | -36.59 | 81.17 | 0.656 | -2.89 | 10.80 | 0.791 |
|  | Alternative drugs | 223.93 | 8.92 | <0.001 | 7.91 | 0.78 | <0.001 | -20.47 | 14.62 | 0.173 | -2.38 | 1.94 | 0.231 |
| **Expenditure (×10^4^ USD)** | Bid-winning drugs | 8971.6 | 739.82 | <0.001 | 429.96 | 64.89 | <0.001 | -4783.3 | 1212.8 | <0.001 | -123.31 | 161.33 | 0.452 |
|  | Non-winning drugs | 6451.49 | 181.42 | <0.001 | -58.94 | 15.91 | <0.05 | -4767.71 | 297.41 | <0.001 | 48.97 | 39.56 | 0.227 |
|  | The branded drug | 9265.64 | 576.32 | <0.001 | 379.11 | 50.55 | <0.001 | -10106.06 | 944.78 | <0.001 | -163.96 | 125.67 | 0.203 |
|  | Generic drugs | 6199.71 | 340.05 | <0.001 | -23.16 | 29.82 | 0.444 | 598.4 | 557.45 | 0.293 | 123.23 | 74.15 | 0.109 |
|  | Alternative drugs | 2924.57 | 87.26 | <0.001 | 47.14 | 7.65 | <0.001 | -466.87 | 143.05 | 0.003 | -58.09 | 19.03 | 0.005 |
| **DDDc (USD)** | Bid-winning drugs | 135.20 | 2.09 | <0.001 | -1.51 | 0.18 | <0.001 | -87.57 | 3.42 | <0.001 | 1.26 | 0.46 | 0.010 |
|  | Non-winning drugs | 11.59 | 0.41 | <0.001 | -0.29 | 0.04 | <0.001 | 0.28 | 0.68 | 0.679 | 0.30 | 0.09 | 0.003 |
|  | The branded drug | 134.72 | 1.93 | <0.001 | -1.39 | 0.17 | <0.001 | -5.07 | 3.16 | 0.121 | 0.53 | 0.42 | 0.219 |
|  | Generic drugs | 11.95 | 0.35 | <0.001 | -0.35 | 0.03 | <0.001 | 2.61 | 0.57 | <0.001 | 0.40 | 0.08 | <0.001 |
|  | Alternative drugs | 17.71 | 0.25 | <0.001 | -0.14 | 0.02 | <0.001 | 0.87 | 0.42 | 0.047 | 0.04 | 0.06 | 0.478 |
